# Genomic Landscape of Systemic Lupus Erythematosus and Lupus Nephritis Globally and the Gaps in Africa

**DOI:** 10.3390/ijms27167383

**Published:** 2026-08-18

**Authors:** Bianca G. Obadic, Jonathan N. Katsukunya, Bianca Davidson, Erika S. W. Jones, Bridget Hodkinson, Robert Freercks, Collet Dandara, Khuthala Mnika

**Affiliations:** 1 Division of Human Genetics, Department of Pathology, Faculty of Health Sciences, Institute of Infectious Disease and Molecular Medicine, University of Cape Town, Cape Town 7925, South Africa; obdbia001@myuct.ac.za (B.G.O.); ktsjon001@myuct.ac.za (J.N.K.); collet.dandara@uct.ac.za (C.D.); 2 Platform for Pharmacogenomics Research and Translation, South African Medical Research Council, Cape Town 7925, South Africa; bianca.davidson@uct.ac.za (B.D.); erika.jones@uct.ac.za (E.S.W.J.); 3 Division of Nephrology & Hypertension, Department of Medicine, Faculty of Health Sciences, University of Cape Town, Cape Town 7925, South Africa; 4 Kidney and Hypertension Research Unit, Department of Medicine, Faculty of Health Science, University of Cape Town, Cape Town 7925, South Africa; 5 Rheumatology, Department of Medicine, Faculty of Health Sciences, University of Cape Town, Cape Town 7925, South Africa; drbridget@gmail.com; 6 Department of Medicine, School of Medicine, Faculty of Health Sciences, Nelson Mandela University, Gqeberha 6031, South Africa; robert.freercks@mandela.ac.za

**Keywords:** Africa, autoimmune disease, genetics, systemic lupus erythematosus, lupus nephritis

## Abstract

Systemic lupus erythematosus (SLE) is a complex autoimmune disease with a strong genetic component, and lupus nephritis (LN) represents one of its most common and severe organ-specific manifestations. This review synthesises current evidence on the genetic architecture of SLE and LN, with a particular focus on identifying shared and distinct genetic susceptibility loci and highlighting knowledge and data gaps in highly burdened African populations. Across studies, most risk loci, including *HLA-DRB1*, *TNFSF4*, *IRF5*, *STAT4*, *TNFAIP3*, *BANK1*, *BLK*, *ITGAM*, and *FcγR2A/FcγR3A*, converge on key immune pathways such as antigen presentation, type I IFN signalling, B-cell activation, and immune complex clearance. The findings support a substantial genomic overlap between SLE and LN, with most variants contributing to systemic immune dysregulation rather than kidney-specific susceptibility. A limited number of loci, including *PDGFRA*, *HAS2*, and *SLC5A11*, have been implicated in renal involvement, while *APOL1* G1/G2 risk variants are associated with renal disease progression and adverse kidney outcomes among individuals of African ancestry. Despite these advances, relatively few loci have been definitively linked to LN independent of SLE, reflecting both biological overlap and limitations in study design. Moreover, the existing literature is heavily skewed toward European, Asian, and admixed populations, with minimal representation of continental African cohorts. Given the high genetic diversity and disproportionate disease burden in African populations, this represents a critical knowledge gap. Improved inclusion of diverse populations, coupled with high-resolution genomic and functional studies, will be essential to refine causal variant identification and enhance understanding of disease mechanisms. Ultimately, insights into population-specific genetic risk may enable earlier identification of high-risk individuals and support the development of precision medicine strategies for SLE, specifically LN.

## 1. Introduction

Systemic lupus erythematosus (SLE) is a highly heterogeneous chronic inflammatory autoimmune disorder characterised by dysregulation of the body’s immune system due to loss of tolerance towards nuclear self-antigens [1]. This leads to the production of autoantibodies which attack the body’s own cells and tissues, and the development of immune complexes. These immune complexes are deposited in the body, triggering widespread inflammation and progressive tissue destruction and organ damage in various parts of the body, including the skin, joints, central nervous system, cardiovascular system, and the kidneys [2,3,4,5]. Lupus nephritis (LN), which is characterised by persistent inflammation in the kidneys, is one of the most common and severe complications associated with SLE. Approximately 50% of individuals diagnosed with SLE will develop LN during their lifetime, typically within 5 years of SLE onset, with many patients developing LN at SLE onset [6,7,8].

The diagnosis of LN is based on a combination of clinical assessment and laboratory tests, including blood and urine analyses [1]. SLE patients who present with a persistent decline in kidney function, proteinuria, haematuria, or active urinary sediment are investigated for LN. To definitively diagnose LN, kidney biopsies are considered the gold standard [6]. The histopathological features identified by the biopsy can then be used to classify LN into six morphological classes, according to the 2018 revised International Society of Nephrology (ISN) and Renal Pathology Society (RPS) classification system [9]. These classes are shown below in Table 1. LN can progress to end-stage kidney disease, significantly increasing the risk of morbidity and mortality compared to those with SLE who do not develop nephritis [3,10,11]. In individuals of African ancestry, more severe histological classes are more common, further contributing to increased morbidity and mortality [6].

The global prevalence of SLE varies significantly across populations, depending on ethnicity, geographic region, and diagnostic criteria [12,13]. However, epidemiological studies have consistently reported a higher disease burden among individuals of non-European ancestry [11,14]. African, Asian, and Hispanic populations have a two- to three-fold increased risk of developing SLE compared to individuals of European descent [15,16]; however, the exact aetiology of these ethnic disparities remains unclear [17]. Furthermore, individuals of African descent frequently present with more severe disease and display higher mortality rates, which have been reported to reach 35%, compared with less than 15% in non-African populations [18,19].

One study conducted by Somers et al. found the age-standardised incidences and prevalences to be 7.9/100,000 person-years and 111.6/100,000 persons in individuals of African ancestry, and only 3.7/100,000 person-years and 47.5/100,000 persons in people of European ancestry, respectively [20]. These differences in prevalence persist even after migration, with people of African genetic ancestry continuing to face a higher risk of developing SLE while residing in North America or Europe compared to those of European ancestry. This indicates that the difference may be attributed to genetic factors rather than solely environmental ones [21]. Despite the substantial burden of LN, with up to 60% of SLE patients developing LN, accurate estimates of its global prevalence remain challenging to obtain. Epidemiological data are lacking for many regions, with Tian et al. reporting that nearly 80% of countries worldwide have insufficient data [11], suggesting that the true burden of LN is likely underestimated in many populations.

**Figure 1:**
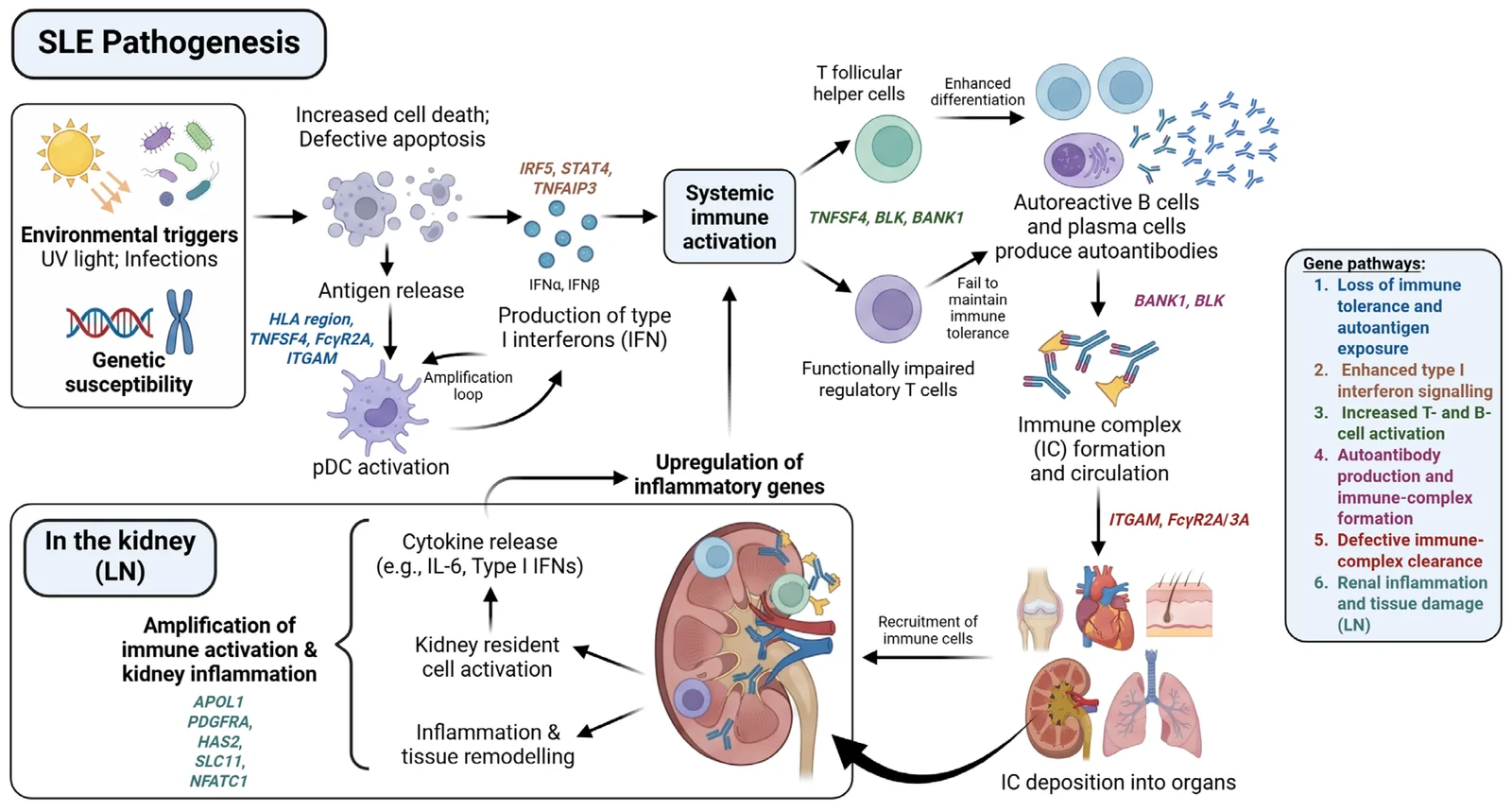
Pathogenesis of SLE and LN. Environmental triggers interact with genetic susceptibility factors to promote increased cell death and defective clearance of apoptotic debris. The resulting release of nuclear autoantigens activates plasmacytoid dendritic cells (pDCs), leading to sustained production of type I IFNs (IFN-α and IFN-β). Type I IFN signalling drives systemic immune activation, promoting production of autoantibodies, which form circulating immune complexes (ICs). Deposition of these ICs in target organs, particularly the kidneys, triggers inflammation, complement activation, and tissue injury characteristic of LN. Selected susceptibility genes associated with key pathogenic stages are indicated. *HLA* = human leukocyte antigen; *TNFSF4* = Tumour Necrosis Factor Superfamily Member 4; *IRF5* = interferon regulatory factor 5; *STAT4* = signal transducer and activator of transcription 4; *TNFAIP3* = tumour necrosis factor-alpha inducible protein 3; *BANK1* = B-cell scaffold protein with ankyrin repeats 1; *BLK* = B-lymphoid tyrosine kinase; *ITGAM* = integrin alpha M; *FcγR =* Fc gamma receptor (created in BioRender. Obadic, B. (2026) https://BioRender.com/7zgzpjt, accessed on 5 August 2026).

The pathogenesis of SLE, with and without LN, is multifaceted, involving a convergence of environmental, immunological, and genetic or epigenetic factors, which collectively disrupt immune homeostasis and promote autoimmunity [2]. Environmental triggers such as ultraviolet light and infections can initiate SLE by promoting increased cell death and impaired clearance of apoptotic debris, resulting in the accumulation of nuclear autoantigens that activate plasmacytoid dendritic cells [22,23]. These cells produce type I IFNs, driving a sustained autoimmune response [2]. Chronic activation of immune cells leads to the production of autoantibodies, which form immune complexes that circulate and deposit in organs. This process can be seen in Figure 1. In LN, immune complexes and activated immune cells, including neutrophils, macrophages, and T cells, accumulate in the glomeruli and tubulointerstitial regions of the kidney, causing inflammation and tissue remodelling [3,5]. Kidney-resident cells such as podocytes, mesangial cells, and tubular epithelial cells also play an active role in LN pathogenesis by releasing cytokines and chemokines, including IL-6 and type I IFNs [3].

These inflammatory mediators amplify immune activation and hence kidney inflammation, with type I IFN signalling upregulating the expression of proinflammatory genes, while cytokines such as IL-6, IL-17, and TNF-α further exacerbate tissue damage [24]. Immune dysregulation in LN is also driven by T follicular helper cells, which enhance B cell differentiation into autoreactive plasma cells, and by functionally impaired regulatory T cells, which fail to maintain immune tolerance. Tissue-infiltrating CD8^+^ T cells and Th17 cells also contribute directly to kidney injury [2].

Family and twin studies have provided strong evidence that there is a genetic contribution to SLE, with monozygotic twins showing a much higher concordance rate (24–56%) compared to dizygotic twins (2–4%) [21,25]. This genetic predisposition has been further elucidated by genome-wide association studies (GWASs) and candidate gene approaches, which have identified numerous susceptibility loci across various populations [25,26,27]. Notably, genes involved in immune regulation, particularly those in the human leukocyte antigen (HLA) region of the major histocompatibility complex (MHC), play a central role in disease susceptibility and pathogenesis [25,28,29,30,31,32]. Additionally, low copy numbers of complement genes within the MHC region have been linked to SLE risk [21]. Genes which regulate apoptosis may also be associated, since defective apoptosis has been shown to be a significant trigger in the development of SLE [22,23,33,34].

While variants within the MHC region remain the strongest risk factors for SLE, studies have highlighted that non-MHC genetic loci play a more decisive role in the transition from systemic autoimmunity to the development of LN [35]. These include genes involved in key pathways, such as IFN signalling, immune complex clearance, lymphocyte activation, and renal fibrosis [17]. Variants in these genes enhance immune cell activation and cytokine production, amplifying inflammation, which eventually culminates into organ damage [25,31]. This offers insight into both pathogenic mechanisms and potential therapeutic targets for SLE and LN. Despite the growing evidence around genetic contributions to disease susceptibility, the function of these implicated genetic factors in disease pathogenesis remains largely unknown. In populations of African ancestry, despite the high disease burden, our knowledge on the genetic contributors is even more limited [36].

This review seeks to provide an overview on what is known about genetic susceptibility to SLE and LN globally, devoting particular attention to the highly burdened African populations. It will evaluate the genomic overlap in SLE and LN, with the goal of distinguishing between variants that contribute specifically to LN susceptibility. It maps out how genetic variation may translate to disease pathogenesis and highlights how insights into population-specific genetic risk factors may inform risk stratification, early identification of high-risk individuals, and targeted prevention strategies at a population level.

## 2. Literature Search Strategy

This narrative review was informed by a structured search of the published literature to provide a comprehensive overview of the genetics of SLE and LN. Literature searches were performed using PubMed, Scopus, and Google Scholar. Additional relevant articles identified through reference list screening were also included. Search terms combined disease-related keywords (“systemic lupus erythematosus”, “SLE”, “lupus nephritis”) with genomic terms such as “genetics”, “GWAS”, “whole exome sequencing”, “whole genome sequencing” and “SNP”, as well as ancestry-related keywords (“Africa”, “African American”, “sub-Saharan Africa”). Where relevant, specific candidate genes frequently implicated in SLE and LN were also included as search terms. Search strategies were adapted appropriately for each database to maximise sensitivity and specificity. Searches were limited to studies published up to May 2026.

The search strategy focused on primary research studies reporting original genetic or genomic data in human SLE and/or LN cohorts. Emphasis was placed on studies investigating susceptibility loci, renal-specific sub-phenotypes, genotype–phenotype associations, and functional implications of identified variants. Particular attention was paid to studies including African populations, given disparities in disease burden and genomic representation. The identified literature was synthesised thematically, with genes grouped according to their functional pathways.

## 3. Results

The literature identified numerous genetic loci commonly reported to be associated with susceptibility to SLE and LN across diverse populations, including European, African American, Asian, Hispanic, admixed African and continental African populations, with greater data available from populations of European, Asian, and African American or admixed African ancestry, highlighting the relative scarcity of genomic data from continental African populations and the lack of replication studies in these populations. The genes discussed are representative and are intended to illustrate key pathogenic pathways with robust evidence for disease susceptibility and current knowledge gaps rather than provide an exhaustive list of all reported SLE/LN susceptibility loci.

The number of published genetic studies in SLE and LN, while growing, remains unevenly distributed across global populations. Table 2 summarises the relative representation of ancestry groups within the available literature and highlights the limited inclusion of continental African populations. In this review, the terms “African American”, “North African”, and “sub-Saharan African” are used according to the populations described in the original studies. The term “continental African” refers specifically to populations residing within Africa and is distinguished from admixed populations of African ancestry where appropriate.

### 3.1. Genetic Variations Associated with SLE and LN

The selected studies reported on genes involved in antigen presentation and adaptive immune activation (*HLA* region, *TNFSF4*), innate immune sensing and the type I IFN pathway (*IRF5*, *STAT4*, *TNFAIP3*), B-cell signalling and autoantibody production (*BANK1*, *BLK*), immune complex clearance (*ITGAM*, *FCGR2A/3A*), and kidney-specific activation (Table 3).

### 3.2. Literature Synthesis

Multiple genetic studies, including large-scale GWAS and trans-ancestral meta-analyses, have identified more than 100 SLE risk loci. The strongest and most frequently replicated associations include the genes found in the MHC locus, particularly the human leukocyte antigen (HLA) class II region. Outside of the MHC region, genes that are commonly implicated are involved in pathways such as antigen presentation and adaptive immune activation, innate immune sensing and the type I IFN pathway, B-cell signalling, autoantibody production, and immune complex clearance [17,73]. Variants in these genes enhance immune cell activation and cytokine production, amplifying inflammation, which eventually culminates into organ damage [25,31]. In addition to these pathways, genes explicitly involved in driving the development of nephritis through processes such as renal fibrosis have been identified. This offers insight into both pathogenic mechanisms and potential therapeutic targets for SLE and LN.

### 3.3. Antigen Presentation and Adaptive Immune Activation

#### 3.3.1. HLA-DR2/DR3

The MHC, found on chromosome 6p21, is one of the most consistently implicated genetic regions in SLE susceptibility. Its primary role is in regulating the immune system by presenting antigens to T cells. In SLE, variation within the MHC can alter peptide binding and presentation, promoting the presentation of self-antigens to autoreactive CD4^+^ T cells, thus amplifying aberrant immune responses and consequently leading to systemic inflammation, driving the disease’s pathogenesis [112].

The highly polymorphic nature of the MHC region together with its extensive linkage disequilibrium (LD) have made identifying precise susceptibility variants a considerable challenge; however, variation within the HLA class II region, and particularly the *HLA-DRB1* locus, which governs antigen presentation to CD4^+^ T cells, has been consistently implicated in SLE susceptibility [46]. Two haplotypes, HLA-DR3 and HLA-DR2, are the two most well-established risk factors for SLE within this region. They are represented by the HLA-DRB1*03:01 allele (DRB1*03:01-DQB1*02:01) and HLA-DRB1*15:01 allele (DRB1*15:01-DQB1*06:02), respectively. These alleles have been shown to be markedly enriched in individuals with SLE compared to healthy controls [43]. Several studies have consistently replicated these associations, particularly in European populations, showing that carriers of these haplotypes have around a two-fold increased risk of SLE [37,39,42,44]. Studies focusing on Asian populations have found similar results [40,45].

Studies in African American ancestry populations have shown less consistent results regarding the influence of *HLA-DR* alleles. Ruiz-Narvaez et al. identified distinct association signals within the HLA region. They reported four SNP signals (rs9271366G>A, rs204890C>T, rs2071349C>G, rs2844580T>C) spanning ~1.7 Mb that were independently associated with increased SLE risk in an African American population. The strongest association signal was for SNP rs9271366G>A, near *HLA-DRB1*, where the G allele conferred an approximately 70% increased risk of SLE [41]. This result is consistent with studies that found the minor G allele of SNP rs9271366G>A to be associated with increased risk of SLE in Asian populations [40,113]. Notably, in this African American cohort, the classical European risk haplotypes (DR2 and DR3) were not significantly associated with disease [41], suggesting that SLE susceptibility in admixed African ancestry populations may be driven by alternative variants within the same region. It stands that susceptibility in continental African populations would differ too.

In contrast to this, trans-ancestral analyses by Hanscombe et al. indicate that the HLA region does remain a major susceptibility locus across populations including African Americans. However, the specific risk alleles may differ. They showed that DR3 represents the strongest signal in European populations, whereas DR2 is more prominent in African American cohorts, reflecting differences in LD structure and haplotype architecture across ancestries [38].

The influence of these haplotypes extends significantly to LN. Several studies have reported that HLA-DR3 in particular confers increased risk of LN in populations of European ancestry [39,43,47,48,49]. Bolin et al. found significant associations between an *HLA-DR3* marker SNP (rs3135394A>G) and increased LN risk in a Swedish population for carriers of the A allele [49]. Similarly, Chung et al. found that, in addition to *DR3*, *DR2* was also enriched in European patients with LN. Using tag SNPs rs9271366G>A (for *DR2*) and rs2187668C>T (for *DR3*), they reported that for individuals carrying the G and T alleles, respectively, LN risk increased by up to 50% [48]. This relationship was further corroborated by Niu et al., who showed that DR3 conferred a higher relative risk of LN than for SLE overall, suggesting that *DR3* carriers may be prone to developing renal manifestations of the disease [50].

#### 3.3.2. TNFSF4

Tumour Necrosis Factor Superfamily Member 4 *(TNFSF4*, also called *OX40),* located on chromosome 1q25, encodes a co-stimulatory ligand, OX40L, expressed on activated antigen-presenting cells and endothelial cells, with its receptor TNFRSF4 (OX40) expressed primarily on activated CD4^+^ T cells [10]. This ligand–receptor pair plays an essential role in T-cell activation and the maintenance of adaptive immunity. Variation within the gene results in increased protein expression, which subsequently drives enhanced T-cell activation, B-cell differentiation, and autoantibody production, positioning the locus as a plausible mediator of autoimmune dysregulation [51,114].

Data reported by Cunninghame-Graham et al. highlighted *TNFSF4* as a candidate gene for SLE susceptibility in European ancestry populations from the UK and a replication cohort from the US [51]. Specifically, they identified an over-transmitted haplotype (G-T-T-T), tagged by the SNPs rs10912580A>G, rs12039904C>T, rs2205960G>T and rs1234317C>T, which was associated with increased risk [51]. Subsequent fine-mapping has confirmed the relevance of this locus across multiple populations [52,53,54,55].

The rs2205960G>T polymorphism is one of the most consistently replicated variants, with the T allele conferring increased SLE risk among Canadian [53], Chinese [40,55,57], and African American [54] populations. These results were supported by another large-scale multi-ethnic study including European, Asian, Hispanic, and African American populations which demonstrated consistent, genome-wide significant association of the rs2205960T allele with increased risk of SLE in all major ancestries [52]. In addition to rs2250960G>T, other commonly implicated variants include the T alleles of rs1234317C>T [52] and rs1234315C>T [40]. These consistent reports may imply that multiple variants within this locus contribute to SLE risk and highlight its global relevance.

Beyond its direct genetic effect, functional analyses suggest potential gene–gene interactions between *TNFSF4* and other known susceptibility genes, including *BLK* and *TNFAIP3*, indicating possible interplay between T-cell and B-cell activation pathways in SLE pathogenesis [55]. The rs2205960T allele in *TNFSF4* has also been linked to increased susceptibility to renal disorder in European populations [58], as well as in a Chinese cohort [59], suggesting a potential role of the *TNFSF4* locus in LN pathogenesis.

#### 3.3.3. PTPN22

The protein tyrosine phosphatase non-receptor type 22 (*PTPN22*) gene, located on chromosome 1p13.3–13.1, encodes the lymphoid tyrosine phosphatase (LYP), a key negative regulator of T-cell receptor signalling that contributes to the maintenance of immune tolerance. Genetic variants within *PTPN22* can disrupt this regulatory pathway, promoting aberrant T-cell activation, autoantibody production, and the loss of self-tolerance that characterise autoimmune diseases.

The rs2476601C>T (c.1693T>C; p.Arg620Trp) variant is the most extensively studied *PTPN22* polymorphism in SLE and represents one of the strongest non-HLA susceptibility variants in European populations. In the first SLE association study of this variant, Kyogoku et al. demonstrated that carriers of at least one rs2476601T allele had a significantly increased risk of developing SLE [64], a finding that has subsequently been replicated across multiple independent European cohorts [39,60,61,62,63].

In contrast, the association between rs2476601C>T and SLE has not been consistently replicated in non-European populations. Studies in African American, Hispanic, and Asian cohorts have generally failed to detect a significant association between 620W and increased SLE susceptibility, largely reflecting the low frequency or absence of the rs2476601T allele in these populations [40,54,63]. However, this does not exclude a role for *PTPN22* in SLE outside European populations. In a large multi-ethnic study, Namjou et al. investigated additional *PTPN22* variants, including rs1217414, rs33996649C>T (c. 788G>A; p.Arg263Gln), rs2488457G>C, and rs3765598C>T. While no significant associations were observed in African American or Asian ancestry cohorts, the rs3765598T allele was significantly associated with SLE in the Hispanic population [63]. Similarly, Tang et al. found no association between R620W and SLE in a Han Chinese cohort but identified the G allele at both rs1217414G>A and rs3811021A>G as protective variants, whereas rs3765598T was associated with increased disease risk [65].

Collectively, these findings demonstrate that although *PTPN22* is an established SLE susceptibility locus, the specific risk variants differ substantially between ancestral populations. The strong association of rs2476601C>T with SLE appears largely restricted to individuals of European ancestry, whereas other *PTPN22* polymorphisms may contribute to disease susceptibility in Hispanic and East Asian populations. These observations underscore the importance of conducting genetic studies across diverse populations to identify ancestry-specific risk variants and improve our understanding of SLE pathogenesis. Currently, significant evidence supporting the role of *PTPN22* in the development of LN specifically is limited, suggesting that its primary contribution is to overall SLE susceptibility rather than kidney involvement [63].

### 3.4. Innate Immune Sensing and Type I Interferon Pathway Genes

#### 3.4.1. IRF5

*IRF5*, located on chromosome 7q32.1, encodes the interferon regulatory factor 5, a central transcription factor within the type I interferon (IFN) pathway and is one of the most consistently reported susceptibility genes for SLE across global populations (Table 2). Functionally, IRF5 regulates the expression of type I IFNs, proinflammatory cytokines, and antigen presentation pathways, thereby influencing both innate and adaptive immune responses. Functional variants in *IRF5* lead to over-expression of IFNs, thus promoting immune dysregulation and autoimmunity [115,116].

The *IRF5* rs2004640G>T polymorphism is one of the most robustly associated variants with SLE, with the T allele conferring increased disease susceptibility. This association was first identified in Finnish and Swedish populations [66] and subsequently replicated in four independent cohorts from the United States, Spain, Sweden, and Argentina [67]. Numerous studies have since confirmed this association in diverse populations, including Europeans [42,69], Asians [74], Hispanics [70], and African Americans and North Africans [71,75], establishing rs2004640G>T as a consistently replicated SLE susceptibility locus. In contrast to these findings, a South Indian population study found no associations between rs2004640G>T and SLE, indicating that, despite the implication of common variants in diverse ancestry groups, there is some level of population specificity for some *IRF5* signals [72].

In addition to rs2004640G>T, the minor alleles (C, C, T and T) of other variants, including rs2070197T>C, rs10488631T>C, rs12539741C>T, and rs3807306G>T [53,68,71,73], have been reported to increase susceptibility to SLE in European and admixed African populations, further supporting the contribution of *IRF5* variation to disease risk across multiple ancestries.

Direct studies investigating the influence of *IRF5* variants on LN susceptibility are limited, but available data suggest a potential role. Qin et al. reported that the T allele of rs2004640G>T was more frequent in Chinese LN patients than in healthy controls, indicating a higher risk of progression to renal disorder. However, these findings did not reflect an association with specific clinical or pathological features, so the variant may not be directly influencing LN development [74]. Further supporting a potential role in LN, Bolin et al. found strong, genome-wide significant signals of association between two *IRF5* SNPs in near perfect linkage, rs2070197T>C and rs10488631T>C, and LN in two independent Swedish cohorts [49]. Hammad et al. also found that the presence of the rs2004640T allele increased LN susceptibility in a North African cohort [75]. These findings indicate that *IRF5* may contribute not only to overall SLE susceptibility but also to the risk of nephritis.

#### 3.4.2. STAT4

Signal transducer and activator of transcription 4 (STAT4) is a transcription factor encoded by the *STAT4* gene located on chromosome 2q32.2–q32.3. *STAT4* is activated primarily by interleukin-12 and type I IFNs, promoting Th1 differentiation, IFN-γ production, and downstream proinflammatory signalling [77]. Its central role in IFN-driven immune activation provides a strong mechanistic basis for involvement in SLE pathogenesis. Functional studies show that mutations in this gene elevate *STAT4* expression, thus amplifying IFN activation, and resulting in an enhanced inflammatory response [76,80]. Genetic studies across diverse global cohorts have identified *STAT4* as a major non-HLA susceptibility locus for SLE. The rs7574865T>G polymorphism is the most consistently associated *STAT4* variant with SLE susceptibility. Remmers et al. reported that the rs7574865T/T genotype was associated with up to a 2.5-fold increased risk of SLE in European populations [76]. This association has been robustly replicated across multiple populations, including European, Asian, African American, and Hispanic cohorts [42,54,77,78,79]. Additional variants that have been associated with increased SLE risk include the G, A, C, and C alleles at rs10181656G>C, rs13017460A>G, rs1517352A>C, and rs3821236G>A, respectively, in European and Asian populations [53,77].

The rs7574865T>G variant has also been strongly linked to the development of nephritis. Taylor et al. demonstrated that the rs7574865T allele was associated with a two-fold increased risk of LN in European American cohorts, with even stronger associations observed in severe nephritis [80]. Bolin et al. corroborated these findings in a Swedish cohort, along with identifying the association between an over two-fold risk of LN and three additional SNPs, rs11889341C>T, rs7568275G>C, and rs7582694C>G, which are found in strong LD [49]. Another study in a Chinese population found further evidence that rs11889341C>T is associated specifically with LN [81].

#### 3.4.3. TNFAIP3 (A20)

The tumour necrosis factor-alpha inducible protein 3 gene (*TNFAIP3*), found on chromosome 6q23.3, encodes A20, a ubiquitin-modifying enzyme that functions as a critical negative regulator of NF-κB signalling, thereby modulating inflammatory responses and preventing excessive immune activation [87]. Dysregulation of A20 activity contributes to prolonged NF-κB signalling, enhanced proinflammatory cytokine production, and loss of peripheral tolerance, all of which are key pathways associated with SLE pathogenesis [10]. This makes it a plausible candidate gene to consider for conferring LN/SLE susceptibility.

The *TNFAIP3* polymorphisms, such as rs5029939C>G and rs2230926T>G (c.380T>G; p.Phe127Cys), have been strongly and consistently linked to SLE susceptibility across multiple diverse populations. These SNPs are found in LD, and among Europeans, individuals who are carriers of the G alleles have an increased disease risk [82]. The role of these SNPs was further confirmed in an independent European cohort [83], as well as studies in Asian populations [84,85]. In addition to these risk alleles, other SNPs including rs6922466A>G and rs13192841G>A have minor alleles G and A, respectively, which are associated with a protective effect against SLE development in Europeans [83].

Studies focused on African American ancestry populations are consistent with these global patterns [86,87]. In addition to the well-established risk variants, rs2230926T>G and rs5029939C>G, African-specific variants including SNP rs5029953G>A, in which the G allele provided a strong risk signal, as well as rs5029941C>T (c.374C>T; p.Ala125Val), a rare minor allele associated with protection from SLE, have been identified [87]. This reinforces the gene’s cross-population relevance.

A comprehensive meta-analysis by Liu et al. reported a significant association between the rs2230926G allele and SLE in Europeans, Asians, and admixed Africans, consolidating the gene’s position as a key component of SLE pathogenesis [117].

There is limited evidence to suggest that *TNFAIP3* variants may also play a role in LN pathogenesis. However, Bates et al. evaluated the associated clinical manifestations of the established SLE risk allele rs5029939C>G in two European cohorts. They found that individuals carrying the G risk allele were twice as likely to develop renal manifestations [88]. Further corroborating the gene’s potential role is the findings in a Japanese study which showed that the effect size of the rs2230926G allele was even larger in individuals with nephritis [84], and Chan et al. showed that this SNP was associated specifically with LN and not SLE without nephritis in their Chinese cohort [81].

### 3.5. B-Cell Signalling and Autoantibody Production

*BANK1*, found on chromosome 4q24, and *BLK*, found on chromosome 8p23.1, encode the B-cell scaffold protein with ankyrin repeats 1 and B-lymphoid tyrosine kinase, respectively. Both genes act as downstream modulators of B-cell receptor signalling and downstream activation pathways. In SLE, B-cell receptor signalling is dysregulated, resulting in exaggerated downstream activation, which promotes the survival of autoreactive B-cells and the production of autoantibodies, thus leading to inflammation [39,89,92,93].

*BANK1* was first implicated in SLE by Kozyrev et al., who reported on variants including rs10516487G>A (c.182G>A; p.Arg61His), rs17266594T>C, and rs3733197G>A (c.1147G>A; p.Ala383Thr) in a Swedish SLE cohort that affect BANK1 protein structure and splicing, thereby enhancing B-cell receptor signalling and contributing to B-cell hyperactivity. They replicated their initial findings in four additional independent European ancestry cohorts from Argentina, Germany, Spain, and Italy, confirming *BANK1* as an SLE risk gene [92]. Since then, the associations of these risk variants with SLE have been replicated in multiple independent populations including European [53,78,89], Asian [57], Hispanic [90], and admixed and North African [54,91] ancestry cohorts. Consolidating evidence that these *BANK1* polymorphisms contribute to SLE susceptibility across diverse ethnic groups was provided by a meta-analysis conducted by Fan et al. [118].

Polymorphisms within the *C8orf13-BLK* locus, including rs13277113G>A, rs2736340C>T, and rs2618476T>C, represent some of the earliest non-HLA GWAS signals for SLE susceptibility and have been consistently replicated across European, Asian, and African ancestry populations. Functionally, the A, T, and C alleles, respectively, are associated with reduced *BLK* expression in B cells, leading to altered B-cell development and signalling thresholds [55].

The rs13277113G>A polymorphism is a key variant at this locus, with the A allele conferring increased susceptibility to SLE. This association has been widely replicated across multiple populations, including European cohorts [39,42,82], Japanese populations [93], Mexican populations [90], as well as African American [54] and North African [91] cohorts, supporting the robustness of this signal. The rs2736340C>T variant has similarly been associated with SLE, with the T allele conferring increased disease risk. This association has been replicated in Mexican populations [90], as well as in Chinese cohorts [55], demonstrating consistency across both admixed and East Asian populations. The C allele of another SNP, rs2618476T>C, has also been observed to increase risk of SLE, with replication observed in Canadian trio studies [53] and additional support from African American cohorts [54], suggesting that multiple variants within the *BLK* region contribute to disease susceptibility, as well as underlining the locus’ cross-population relevance.

Individually, *BLK* and *BANK1* confer a modest SLE risk, but evidence suggests that these two genes may interact to affect B-cell function. Studies in Chinese cohorts confirmed *BLK* rs2736340T allele and *BANK1* rs10516487G allele associations with increased SLE risk and, importantly, observed a significant interaction between *BLK* and *BANK1* genotypes, suggesting that they function within the same B-cell pathway [119]. This genetic interaction was also observed in a Latin American cohort between *BLK* rs2736340C>T and rs13277113G>A, and *BANK1* R61H and A383T [90].

Direct evidence linking these genes specifically to LN is currently limited. However, some studies suggest that genetic variation *BANK1* and *BLK* could play a role. According to Alonso-Perez et al., European patients with LN who were carriers of the *BLK* rs13277113A allele had an increased susceptibility to LN [94]. This was corroborated by Di et al., who found that the same SNP was associated with renal disorder in Chinese SLE patients [95], implying a potential role in LN. Additionally, Chan et al. found the rs2736340T allele to be significant in conferring risk of LN in Chinese SLE patients [81]. In contrast, other studies have reported no significant association with LN specifically [42,58]. For *BANK1*, Bolin et al. found evidence of associations between several highly linked SNPs in *BANK1*, with the strongest association being for the A allele of rs4699261G>A, and increased risk of LN in European cohorts [35]. However, other studies have failed to detect any association [58].

### 3.6. Effector Phase and Immune Complex Clearance Genes

#### 3.6.1. ITGAM (CD11b)

*ITGAM*, located on chromosome 16p11.2, encodes integrin alpha M, known as CD11b, a subunit of the complement receptor 3 (CR3) expressed on antigen-presenting cells. CD11b mediates phagocytosis, leukocyte adhesion, complement receptor activity, and clearance of immune complexes. These functions are central to maintaining immune homeostasis. As such, genetic variation in *ITGAM* has been shown to play a role in SLE [97].

The *ITGAM* gene was first identified as a major SLE susceptibility locus in a large multi-ancestry GWAS, where the A allele of rs1143679G>A (c.230G>A; p.Arg77His) was shown to confer substantial disease risk first in a European cohort and then with replication in two independent African American cohorts [97]. This missense variant alters the CD11b extracellular domain, impairing ligand binding and reducing phagocytic clearance of immune complexes and apoptotic cells, thereby permitting inflammatory responses.

This importance of *ITGAM* has been reaffirmed in subsequent independent European cohorts [39,42,78], with the identification of an additional SNP, rs9888739C>T, where the T allele conferred increased disease risk, by Harley et al., as well as in admixed African ancestry cohorts [54,98]. Han et al. confirmed the multi-ethnic relevance with their findings that rs1143679G>A was associated with increased SLE risk in European, Hispanic, and African American populations [56]. Despite not finding an association of rs1143679G>A in Asian populations, other studies have demonstrated that this variant, as well as the T allele of rs1143683C>T (c.2576C>T; p.Ala859Val), another non-synonymous *ITGAM* variant, does increase SLE susceptibility in Asians [79,99], confirming robust global association and relevance of *ITGAM* to SLE.

There is also evidence that supports the role of *ITGAM* in the development of nephritis. Studies involving both European [49,58,100] and Asian [99] cohorts have reported that rs1143679G>A is significantly associated with LN, with carriers of the A allele having almost twice the risk of developing renal disease in addition to associations with discoid rash and serological abnormalities. These results demonstrate a clear genotype–phenotype effect, implicating impaired CD11b function in renal immunopathology, and they support the notion that *ITGAM* influences the broader clinical expression of SLE beyond general susceptibility.

#### 3.6.2. FcγR2A and FcγR3A

Fc gamma receptors (FcγRs) regulate antibody-mediated immune responses by binding to the Fc region of immunoglobulin G (IgG) and modulating effector pathways such as phagocytosis, immune complex clearance, and activation of inflammatory cascades [10]. Given the central role of immune complex deposition in SLE, polymorphisms within two low-affinity activating-type receptors, *FcγR2A* and *FcγR3A*, which can influence immune complex clearance efficiency, have been extensively examined as candidate susceptibility loci [102].

*FCGR2A* SNP rs1801274A>G (c.500A>G; p.His167Arg) has co-dominantly expressed allelic variants, 131R and 131H, which differ in their binding affinity for IgG subclasses. The 131H allele exhibits higher affinity for IgG2, facilitating more efficient immune complex clearance, whereas the 131R variant demonstrates reduced binding capacity.

Early evidence from Salmon et al. demonstrated that the 131H allele was significantly underrepresented in SLE cases compared to controls in an African American cohort, suggesting that it confers a protective effect against SLE susceptibility compared to the 131R allele [102]. Subsequent studies across European [103], Asian [45,104], African American [105], and Hispanic [101] populations have corroborated these findings, reporting increased frequency of the alternate 131R allele among SLE patients compared to controls, and associations with nearly double the disease risk in 131R/R homozygotes. Importantly, these data seem to suggest that the impact of *FCGR2A* variation has more of an association with specific disease sub-phenotypes, particularly LN, with several studies reporting the overrepresentation of the 131R allele in LN patients compared to SLE-only patients [102,103,105,106].

In an African ancestry context, Zidan et al. reported a significant association between the 131R allele and renal involvement in Egyptian SLE patients [107]. Similarly, recent work by Radouani et al. in an African Caribbean cohort demonstrated that the *FCGR2A* 131R allele was associated specifically with LN, but not non-renal SLE, further suggesting a role in modulating disease phenotype rather than initial SLE risk [108]. Not only do these data suggest that FcγR risk profiles are clinically relevant in LN susceptibility, but they also indicate that there is particular relevance of *FCGR2A* variants within African ancestry populations. In contrast to these findings, not all studies have replicated these associations, with some finding no significant relationship between *FCGR2A* R131H and LN susceptibility in European, Asian, Hispanic, or African American cohorts, highlighting ongoing inconsistency in the literature [58,109].

Polymorphisms in *FCGR3A* SNP rs396991A>C (c.526T>G; p.Phe176Val), particularly the presence of the low-binding affinity 158F allele, have been widely implicated in increased SLE susceptibility across multiple ethnic groups, including African American, European, Asian, and Hispanic populations [101,105,108,110,111]. Several of these studies further suggest that these variants may be particularly relevant to LN susceptibility. For example, Seligman and colleagues reported that individuals of European ancestry homozygous for 158F had an increased risk of developing LN, although this association was not observed in Hispanic, Asian, or African American populations [109]. In agreement with this, an independent Chinese population study found that while the 158F allele was associated with increased risk of SLE, its effect did not extend to conferring LN risk [111].

Edberg et al. showed that the low IGg binding 158F allele was associated with an increased susceptibility to renal involvement in European and African American cohorts [105]. Corroborating this, Dong et al. showed that in African Americans, individuals carrying the 158F allele had an increased LN risk; however, they found that this effect was absent in European Americans [110]. Mechanistically, the low-affinity *FcγR2A*-131R and *FcγR3A*-158F alleles may impair immune complex clearance or alter inhibitory/activating receptor signalling thresholds, facilitating deposition of pathogenic IgG complexes in the body, thus facilitating an autoimmune response [102].

### 3.7. Kidney-Specific Susceptibility Loci

In addition to susceptibility loci shared with SLE, a limited number of genetic variants have been reported to be more specifically associated with kidney involvement and the development of LN. These include *APOL1* [120,121]; *PDGFRA*, *HAS2*, *SLC5A11*, and *ID4* [48]; *ADCYAP1* [122]; *SPSB1* and *RAET1-AS* [123]; *HGF*, *BACH2*, *SOX1*, and *TTC28* [124]; as well as *ZNF546*, *TRIM15*, *TRIM10*, *TTC34*, and *NFATC1* [17]. These loci are thought to contribute to the transition from systemic autoimmunity to organ-specific renal injury, highlighting biological pathways involved in kidney inflammation, fibrosis, and tissue damage, as reported in Table 3, and illustrated by Figure 1. *APOL1*, located on chromosome 22, represents a distinct category of ancestry-associated renal-specific risk and is one of the most established kidney-specific risk loci. The G1 missense variants and G2 6 bp deletion have been strongly associated with several progressive non-diabetic forms of kidney disease, including LN-associated chronic kidney disease (CKD) [125]. Individuals carrying any two of these *APOL1* risk alleles have an approximately two-fold risk of developing CKD [126,127]. These variants are almost exclusively found in individuals of recent African ancestry, having been positively selected because they confer protection against Trypanosoma brucei infection, while remaining largely absent in European populations [128]. In African American patients with LN, high-risk *APOL1* genotypes are associated with faster progression to end-stage kidney disease and a shorter time to kidney failure, likely through mechanisms involving podocyte injury, mitochondrial dysfunction, inflammasome activation, and interactions of *APOL1* variants with downstream IFN pathways [120]. A recent study by Patel et al. confirmed that African ancestry SLE patients who possess the high-risk G1 and G2 alleles have higher odds of developing nephritis compared to individuals without the high-risk genotypes [121]. Collectively, these findings indicate that *APOL1* acts primarily as a modifier of renal susceptibility, severity, and progression rather than as a determinant of overall SLE susceptibility.

Although these loci represent promising candidates for LN-specific susceptibility, apart from *APOL1*, most have not reached conventional genome-wide significance or been consistently replicated across independent studies and diverse ancestral populations. Consequently, their designation as LN-specific loci should be interpreted cautiously until further genetic, functional, and trans-ancestral validation studies are performed.

**Table 3:** Kidney-specific and LN-enriched loci.

Gene	Locus	Lead SNP	Variant Function	Proposed Effect on LN Development	Population Studied	Reference
***APOL1***	22q12.3	G1 (rs73885319A>G p.Ser342Gly rs60910145T>G p.Ile384Met); G2 (rs71785313 p.Asn388_Tyr389del)	Missense variants (G1) and 6 bp deletion (G2) alter APOL1 function	African-specific; high-risk genotypes (two risk alleles) ↑ susceptibility to LN and progression to ESKD.	African American; continental African	[120,121,126,129]
***PDGFRA***	4q11–q13	rs1364989	Intergenic variant; ↓ PDGFRα expression	↓ PDGFRα signalling may impair renal tissue repair and extracellular matrix homeostasis, increasing susceptibility to immune-mediated kidney injury.	European	[48]
***HAS2***	8q24.12	Not specified	Likely regulatory	May alter hyaluronan synthesis, promoting extracellular matrix remodelling, renal inflammation, and fibrosis.	European	[48]
***SLC5A11***	16p12	Not specified	Likely regulatory	Proposed to influence TNF-α- and PDCD1-mediated inflammatory signalling, ↑ apoptosis, and inflammation.	European	[48]
***ID4***	6p22	Not specified	Function unknown	Associated with LN susceptibility, although the biological mechanism remains unclear.	European	[48]
***ADCYAP1***	18p11.32	rs12606116	Intergenic variant near *ADCYAP1*	↓ d renal *ADCYAP1* expression may ↓ PACAP-mediated protection against oxidative stress, inflammation, and tubular cell death.	Han Chinese	[122]
***SPSB1***	Not reported	rs1294028	Regulatory variant; protective	↑ TGF-β and IFN-α signalling, reducing renal inflammation and fibrosis.	Taiwanese	[123]
***RAET1-AS***	Not reported	rs17079029	Variant within *RAET1* gene cluster	May alter NKG2D ligand-mediated NK cell activation, contributing to impaired immune surveillance and renal injury.	Taiwanese	[123]
***HGF***	Not reported	rs1025129	Upstream regulatory variant	May alter *HGF* expression, disrupting HGF/c-Met signalling involved in podocyte repair, tissue regeneration, and the balance with profibrotic TGF-β signalling.	Taiwanese	[124]
***BACH2***	Not reported	rs80282109	Putative splice/regulatory variant	May alter *BACH2* expression and B-cell differentiation, impairing immune tolerance and promoting autoimmunity.	Taiwanese	[124]
***SOX1***	Not reported	rs516119	Upstream regulatory variant; protective	Mechanism remains uncertain but may influence immune regulatory pathways.	Taiwanese	[124]
***TTC28***	Not reported	rs134545	Intronic variant	May influence gene splicing or expression, potentially affecting immune cell proliferation and inflammatory cell migration.	Taiwanese	[124]
***ZNF546***	Not reported	Gene-based association	Gene-level association	Biological mechanism remains unclear.	South European	[17]
***TRIM15***	Not reported	Gene-based association	Gene-level association	Biological mechanism remains unclear.	South European	[17]
***TRIM10***	Not reported	Gene-based association	Gene-level association	Biological mechanism remains unclear.	South European	[17]
***TTC34***	Not reported	Gene-based association	Gene-level association	Biological mechanism remains unclear.	Hispanic	[17]
***NFATC1***	Not reported	rs8091180	Intronic/regulatory variant	May alter NFAT signalling involved in T-cell activation and immune regulation; largest effect observed in African Americans.	Multi-ethnic (European, Hispanic, African American, East Asian)	[17]
***NFATC1***	Not reported	rs68734	Fine-mapped regulatory variant	Stronger association than rs8091180 in African Americans, suggesting a population-specific causal variant influencing immune activation.	African American	[17]

*APOL1* = apolipoprotein L1; *PDGFRA* = platelet-derived growth factor receptor-alpha; *HAS2* = hyaluronan synthase 2; *SCL5A11* = sodium/glucose cotransporter family member 5A11; *ID4* = inhibitor of DNA binding 4; *ADCYAP1* = adenylate cyclase-activating polypeptide 1; *SPSB1* = SPRY domain-containing SOCS box protein 1; *RAET1* = retinoic acid early transcript 1; *HGF*= hepatocyte growth factor; *BACH2*= BTB domain and CNC homologue 2; *SOX1*= SRY-box transcription factor 1; *TTC28* = tetratricopeptide repeat domain 28; *ZNF546* = zinc finger protein 546; *TRIM15* = tripartite motif-containing protein 15; *TRIM10* = tripartite motif-containing protein 10; *TTC34* = tetratricopeptide repeat domain 34; *NFATC1* = nuclear factor of activated T cells, cytoplasmic 1; ↑ = increased/increasing; ↓ = decreased/decreasing.

## 4. Discussion

The aim of this review was to synthesise existing knowledge on the genetic architecture of SLE and LN, with particular emphasis on highlighting loci that may contribute to renal involvement and highlight the relevance of these findings in highly burdened African populations. A consistent finding across the reviewed literature is that the majority of established SLE susceptibility loci converge on key immunological pathways in which variation may lead to dysregulation and loss of self-tolerance.

For example, *HLA-DRB1* and *TNFSF4* are primarily involved in processes such as antigen presentation and activation of adaptive immune responses [51,112]. *HLA-DRB1* remains the strongest genetic contributor to SLE susceptibility, although considerable allelic heterogeneity exists across populations due to differences in LD structure [41], which complicates the identification of causal variants. However, substantial evidence highlights the relevance of two haplotypes, HLA-DR2 and HLA-DR3, as global risk factors for both SLE and progression to LN. The disparities seen emphasise the necessity of trans-ancestral analyses to pinpoint true population-specific causal variants [38]. The global replication of *TNFSF4* variants, particularly rs2205960G>T (Table 2), affirms its relevance as both an SLE and LN susceptibility locus.

*IRF5* and *STAT4*, which play major roles in IFN-driven immune activation, have been identified as some of the most important non-HLA susceptibility loci. In particular, the *IRF5* rs2004640G>T SNP and *STAT4* rs7574865T>G SNP show the most consistent associations with SLE susceptibility in multiple diverse populations (Table 2). Furthermore, our findings indicate that *STAT4* rs7574865T>G not only contributes to overall SLE susceptibility but may also predispose to more severe renal phenotypes, further supporting the role of this locus in disease susceptibility [80]. Limited evidence also links variants within *IRF5* to LN susceptibility [74]. Studies which have focused on populations of African ancestry have not evaluated the contribution of *STAT4* or *IRF5* specifically to LN. However, given the strong associations between these loci and SLE in both admixed and continental African populations, combined with the disproportionately high prevalence and severity of LN in these groups, it is plausible to suggest that *IRF5* and *STAT4* variants may contribute to LN pathogenesis. However, direct LN-focused genetic studies, particularly in continental African cohorts, remain a clear gap in the literature.

Similarly, *TNFAIP3*, a key negative regulator of NF-κB signalling, has effectively been established as an SLE risk locus. Emerging evidence suggests that both shared and ancestry-specific variants contribute to SLE risk, with polymorphisms, such as rs5029939C>G and rs2230926T>G (F127C), showing global relevance (Table 2). African-specific variants were also identified in African American cohorts [87]; however, comprehensive continental African genomic studies remain lacking. While limited evidence linking *TNFAIP3* to LN susceptibility exists, given the relevance of NF-κB activity in renal inflammation, *TNFAIP3* variants are also biologically plausible contributors to the development of nephritis.

Other loci highlighted in this review further support the central role of B-cell dysregulation in SLE and LN pathogenesis. The associations between SLE and variants in *BANK1* and *BLK*, both involved in B-cell receptor signalling, have been replicated across ancestries (Table 2), implying that *BANK1* and *BLK* are broadly relevant susceptibility loci. However, differences in LD patterns between populations, particularly the greater haplotype fragmentation observed in African genomes, may influence which proxy SNPs best capture the causal variants [54]. Consequently, direct fine-mapping studies in African cohorts are essential to clarify the functional variants underlying these associations. While not yet universally replicated as LN-specific loci, considering the role of *BANK1* and *BLK* in pathways driving autoantibody production and immune complex formation, and their previously documented associations with renal manifestations of SLE [35,94], further targeted LN-specific analyses should be considered.

Among the reviewed loci, genes involved in immune complex clearance are commonly implicated. *ITGAM* represents one of the most robustly replicated and mechanistically characterised genetic contributors to SLE (Table 2). Associations between SLE and the missense variant rs1143679G>A (Arg77His), in particular, have been reported across multiple ancestries, with several studies also linking the variant to increased risk of LN [58,100]. The functional effects of this variant, which results in impaired ligand binding and reduced phagocytotic clearance of ICs, directly implicate it in processes central to the development of autoimmunity. Despite this strong global evidence, high-resolution mapping of the *ITGAM* locus in continental African populations remains limited, highlighting an important area for future investigation.

Variants in Fc gamma receptor genes (*FCGR2A* and *FCGR3A*) also provide insight into mechanisms underlying renal involvement in SLE. These polymorphisms influence the affinity of Fc receptors for IgG, thereby affecting immune complex clearance [10]. Evidence suggests that while these variants may not consistently drive overall SLE susceptibility across populations, they can modify disease severity and increase the likelihood of renal complications, thus influencing the transition from systemic autoimmunity to organ-specific manifestations such as LN [102,103,105,106].

While significant progress has been made in identifying SLE risk loci, the roles of many identified genetic variants remain poorly understood, particularly in LN [130]. Table 3 highlights select loci that may be important in LN. Of particular interest is *APOL1*, which is shown to increase the risk of ESKD in LN patients, particularly in populations of African ancestry [120]. Overall, the available evidence suggests that genuinely kidney-specific genetic effects in SLE remain comparatively uncommon and less well replicated than shared SLE susceptibility loci. The loci that have been identified have been implicated in renal inflammation and fibrosis [17,48,124]. The predominance of findings from European and Asian cohorts also limits their generalisability and transferability to other populations, highlighting the need for well-powered LN versus non-renal SLE studies in continental African and other underrepresented populations.

Taken together, these findings highlight the difficulty in clearly separating the genetic basis of SLE from that of LN. Because LN occurs as a clinical manifestation of SLE, many genetic studies do not distinguish between the two phenotypes, often analysing them collectively or using LN as a secondary outcome. As a result, relatively few loci have been conclusively identified as LN-specific susceptibility variants [3]. However, given the cause-and-effect relationship between the two conditions, it is plausible to hypothesise that the genetics of LN likely reflect a combination of genes that contribute to overall SLE susceptibility through immune dysregulation, alongside additional genetic or environmental factors that specifically influence kidney function and increase the risk of developing LN [10]. Lanata et al. reported that many of the strongest LN-associated variants were established CKD risk loci rather than classical SLE susceptibility loci [17]. These findings support the concept that progression from systemic autoimmunity to renal involvement is influenced not only by genes regulating immune dysregulation but also by kidney-specific susceptibility pathways. Furthermore, they provide evidence that risk loci differ between ancestries, which emphasises the need for population-specific studies [17].

A major limitation identified throughout the literature is the underrepresentation of continental African populations in genomic studies of SLE and LN. Most genomic studies have been conducted in populations of non-African ancestry, including admixed American, European, and Asian populations, or admixed African American populations [54,131,132]. This leaves substantial gaps in the knowledge on continental African populations. This lack of representation is particularly concerning given the disproportionately high burden and severity of disease observed among individuals of African ancestry [19,133]. Due to differences in allele frequencies, LD patterns, and the high level of diversity in African populations [134], it follows that the prevalence and impact of these identified genetic factors may differ in African populations, and as such, previous findings may not be applicable across different populations [7,16,54]. *PTPN22* represents a prime example of the limited transferability of genetic associations across ancestral populations, as the lack of reproducibility of associations with SLE likely reflects differences in genetic architecture, including the extremely low frequencies of the rs2476601T allele in some populations (Table S1). This limited transferability also extends to polygenic risk scores (PRSs), which aggregate the effects of multiple genetic variants to estimate disease susceptibility. Studies have found positive correlations between PRS and disease severity, highlighting its potential use as a predictive tool for disease manifestations [135,136]. PRSs have been developed predominantly from European ancestry cohorts and, as such, generally demonstrate reduced predictive performance in individuals of African ancestry and therefore have limited clinical utility [137,138]. The limited number of genetic studies that have included continental African ancestry populations suggests that both shared global risk variants and unique ancestry-specific alleles contribute to disease susceptibility; however, the full effect of genetic ancestry on SLE and LN susceptibility is yet to be elucidated [96]. This highlights the importance of conducting population-specific research [139].

Another notable challenge is that much of the available literature is relatively dated, with many foundational association studies conducted during the early era of genome-wide association studies. More recent large-scale GWAS and trans-ancestral meta-analyses have considerably expanded the number of known SLE susceptibility loci [140]. However, many of these associations require further validation across diverse populations, particularly continental African cohorts, underscoring the continued need for inclusive genomic studies. Furthermore, inconsistencies between studies, particularly when examining the same populations, may reflect methodological differences such as limited sample sizes, heterogeneous case definitions, or inadequate correction for population stratification [141]. Additionally, while these studies have successfully identified numerous susceptibility loci, many of the associated variants occur within non-coding regions of the genome, making their biological consequences difficult to interpret. Such variants may influence disease risk through diverse regulatory mechanisms that alter gene expression, including effects on enhancer activity, transcription factor binding, chromatin structure, and RNA splicing, but ultimately their roles in disease pathogenesis remain incompletely characterised [142,143]. These discrepancies highlight the need for well-powered replication studies and integrative approaches that combine genetic and functional data to better define causal mechanisms.

From a broader perspective, understanding the genetic basis of SLE and LN has important implications for public health. Identifying population-specific risk variants may contribute to improved risk stratification and prediction and earlier identification of individuals at increased risk of severe SLE manifestations such as LN [144]. In highly burdened populations, particularly those in Africa where access to specialised care may be limited, such knowledge could inform targeted screening strategies and enable earlier clinical intervention. Moreover, elucidating the molecular pathways underlying disease susceptibility may facilitate the development of targeted therapies and precision medicine approaches tailored to specific patient populations [145].

This review highlights both shared and population-specific genetic associations while identifying important knowledge gaps, particularly in continental African populations, that warrant further investigation. Nevertheless, several limitations should be acknowledged. As a narrative review informed by a structured literature search, we did not intend to provide a systematic or exhaustive synthesis of all genetic loci associated with SLE and LN. Instead, representative genes with well-established evidence and relevance to key pathogenic pathways were selected to illustrate the current understanding of disease susceptibility and the existing gaps in continental African genomic research. Consequently, several additional loci implicated in SLE and LN, including recently identified susceptibility genes that have not yet been validated and rare or monogenic causes of lupus, were beyond the scope of this review. As the field continues to evolve, future reviews or meta-analyses incorporating emerging genomic discoveries and larger multi-ancestry studies will further refine our understanding of the genetic architecture of SLE and LN.

## 5. Conclusions

Current evidence indicates that many of the genetic loci associated with LN represent extensions of the broader immunological pathways underlying SLE. Many studies fail to distinguish between SLE and variants that contribute to specific sub-phenotypes such as LN. Consequently, the genetic factors that predispose specifically to LN are yet to be fully understood. While several key susceptibility genes have been consistently replicated across multiple populations, the available literature remains heavily skewed toward European, Asian, and North American populations, leaving major gaps in understanding the genetic determinants of disease in continental African populations. Despite this strong global evidence, African-specific high-resolution mapping remains limited. Given differences in LD structure and allele frequencies, dedicated African genomic studies could uncover additional population-specific variants or refine causal interpretation. Addressing these gaps through large-scale, population-inclusive genomic studies will be essential for refining our understanding of disease mechanisms and translating genetic discoveries into clinically meaningful strategies for risk prediction, prevention, and treatment.

## Figures and Tables

**Table 1:** The 2018 ISN/RPS lupus nephritis classification [9].

Class	Description	Lesion Characterisation
I	Minimal mesangial LN	Mild; tracked via immune deposits
II	Mesangial proliferative LN	Pure mesangial hypercellularity or matrix expansion
III	Focal LN	Active and/or chronic lesions; <50% glomeruli affected
IV	Diffuse LN	Active and/or chronic lesions; ≥50% glomeruli affected
V	Membranous LN	Subepithelial deposits; can coexist with Class III or IV
VI	Advanced sclerosing LN	≥90% globally sclerosed glomeruli; irreversible damage

**Table 2:** Genes associated with shared SLE/LN susceptibility, and corresponding SNPs.

Gene	SLE-Associated SNP	Studied Population	LN-Associated SNP	Studied Population	Associated Phenotype
*HLA-DRB1*	DR2 and DR3 haplotypes	EU, AA, AS, HI [37,38,39,40,41,42,43,44,45,46]	DR2 and DR3 haplotypes	EU [47,48,49,50]	Shared SLE/LN
*TNFSF4*	rs2205960G>T rs10912580A>G rs12039904C>T rs1234317C>T rs1234315C>T	EU, AA, AS, HI [51,52,53,54,55,56,57]	rs2205960G>T	EU, AS [58,59]	Shared SLE/LN
*PTPN22*	rs2476601C>T (Arg620Trp) rs33996649C>T (Arg263Gln) rs2488457G>C rs3765598C>T Protective: rs1217414G>A rs3811021A>G	EU, AS, HI [39,60,61,62,63,64,65]	Not reported	Not reported	SLE-specific
*IRF5*	rs2004640G>T rs10488631T>C rs3807306G>T rs2070197T>C rs12539741C>T	EU, AS, HI, AA, NA [42,53,66,67,68,69,70,71,72,73]	rs2004640G>T rs2070197T>C rs10488631T>C	EU, AS, NA [49,74,75]	Shared SLE/LN
*STAT4*	rs7574865T>G rs10181656G>C rs13017460A>G rs1517352A>C rs3821236G>A	EU, AS, AA, HI [39,42,53,54,56,76,77,78,79]	rs7574865T>G rs11889341C>T rs7568275G>C rs7582694C>G	EU [49,80,81]	Shared SLE/LN
*TNFAIP3*	rs5029939C>G rs2230926T>G (Phe127Cys) rs5029953G>A Protective: rs6922466A>G rs13192841G>A rs5029941C>T (Ala125Val)	EU, AS, AA, NA [53,55,82,83,84,85,86,87]	rs5029939C>G rs2230926T>G	EU [81,84,88]	Shared SLE/LN
*BANK1*	rs10516487G>A (Arg61His) rs17266594T>C rs3733197G>A (Ala383Thr)	EU, AS, HI, AA, NA [53,54,57,78,89,90,91,92]	rs4699261G>A	EU [35]	Shared SLE/LN
*BLK*	rs13277113G>A rs2736340C>T rs2618476T>C	EU, AS, HI, AA, NA [39,42,53,54,55,78,82,90,91,93]	rs13277113G>A rs2736340C>T	EU, AS [81,94,95,96]	Shared SLE/LN
*ITGAM*	rs1143679G>A (Arg77His) rs9888739C>T rs1143683C>T (Ala859Val)	EU, AS, AA [39,54,56,78,79,97,98]	rs1143679G>A	EU, AS [49,58,99,100]	Shared SLE/LN
*FCGR2A*	rs1801274A>G (His167Arg)	EU, AS, AA, HIS [45,101,102,103,104,105]	rs1801274A>G	EU, AA, NA, CA, BR [102,103,105,106,107,108,109,110]	Shared; LN-enriched
*FCGR3A*	rs396991A>C (Phe158Val)	EU, AS, HI, CA [101,105,111]	rs396991A>C	EU, AA [45,101,103,105,108,109,110,111]	Shared; LN-enriched

EU = European; AA = African American; AS = Asian; HI = Hispanic; NA = North African (Egyptian); CA = African Caribbean; BR = Brazilian; *HLA* = human leukocyte antigen; *TNFSF4* = Tumour Necrosis Factor Superfamily Member 4; *PTPN22* = protein tyrosine phosphatase non-receptor 22; *IRF5* = interferon regulatory factor 5; *STAT4* = signal transducer and activator of transcription 4; *TNFAIP3* = tumour necrosis factor-alpha inducible protein 3; *BANK1* = B-cell scaffold protein with ankyrin repeats 1; *BLK* = B-lymphoid tyrosine kinase; *ITGAM* = integrin alpha M; *FcγR* = Fc gamma receptor.

## Data Availability

No new data were created or analysed in this study. Data sharing is not applicable to this article.

## Supplementary Materials

The following supporting information can be downloaded at https://www.mdpi.com/article/10.3390/ijms27167383/s1.

## Abbreviations

The following abbreviations are used in this manuscript:

ADCYAP1	Adenylate cyclase-activating polypeptide 1
APOL1	Apolipoprotein L1
BACH2	BTB domain and CNC homologue 2
BANK1	B-cell scaffold protein with ankyrin repeats 1
BLK	B-lymphoid tyrosine kinase
CKD	Chronic kidney disease
ESKD	End-stage kidney disease
FcγR	Fc gamma receptor
GWAS	Genome-wide association study
HAS2	Hyaluronan synthase 2
HLA	Human leukocyte antigen
IC	Immune complex
ID4	Inhibitor of DNA binding 4
IFN	Interferon
IRF5	Interferon regulatory factor 5
ISN	International society of nephrology
ITGAM	Integrin alpha M
LN	Lupus nephritis
MHC	Major histocompatibility complex
NFATC1	Nuclear factor of activated T cells, cytoplasmic 1
PDGFRA	Platelet-derived growth factor receptor-alpha
PRS	Polygenic risk score
PTPN22	Protein tyrosine phosphatase non-receptor type 22
RAET1	Retinoic acid early transcript 1
RPS	Renal pathology society
SCL5A11	Sodium/glucose cotransporter family member 5A11
SLE	Systemic lupus erythematosus
SNP	Single nucleotide polymorphism
SOX1	SRY-box transcription factor 1
SPSB1	SPRY domain-containing SOCS box protein 1
STAT4	Signal transducer and activator of transcription 4
TNFAIP3	Tumour necrosis factor-alpha inducible protein 3
TNFSF4	Tumour Necrosis Factor Superfamily Member 4
TRIM10	Tripartite motif-containing protein 10
TRIM15	Tripartite motif-containing protein 15
TTC34	Tetratricopeptide repeat domain 28
ZNF546	Zinc finger protein 546
