# Supplementary material for: Genomic Landscape of Systemic Lupus Erythematosus and Lupus Nephritis Globally and the Gaps in Africa
Source: Int J Mol Sci. 2026 Aug 18;27(16):7383. doi: 10.3390/ijms27167383 (PMC13513728; doi:10.3390/ijms27167383)
Supplement: Supplementary file 1 [file ijms-27-07383-s001.zip › ijms-4470031-supplementary.pdf]

Table S1. List of SNPs of identified in the literature search, and functional effect of variants

SNP	Alternative name	Variant allele	Functional Effect of Variant Allele	Frequency of variant allele in African populations
HLA-DRB1				
rs9271366G>A	NC_000006.11:g.32586854G>A	G	Regulatory; ↑ HLA class II expression	0.14
rs204890C>T	NC_000006.11:g.32085598C>T	C	Regulatory; (tag SNP)	0.91
rs2071349C>G	NC_000006.12:g.33075743C>G	C	↑ HLA class II expression	0.04
rs2844580T>C	NC_000006.11:g.31333303T>C	C	Regulatory; (tag SNP)	0.26
rs3135394A>G	NC_000006.11:g.32408497A>G	A	↑ HLA-DRA expression	0.98
rs2187668C>T	NC_000006.11:g.32605884C>T	T	↑ HLA class II expression (via DR3/DQ2 haplotype)	0.07
TNFSF4				
rs10912580A>G	NC_000001.10:g.173256550A>G	G	Enhancer; ↑ *TNFSF4* expression	0.12
rs12039904C>T	NC_000001.10:g.173212273C>T	T	Intronic regulatory; ↑ *TNFSF4* expression	0.05
rs2205960G>T	NC_000001.10:g.173191475G>T	T	↑↑ TNFSF4 expression	0.05
rs1234317C>T	NC_000001.10:g.173187775C>T	T	↑ TNFSF4	0.09
rs1234315C>T	NC_000001.10:g.173178463C>T	T	Intronic; ↑ TNFSF4	0.78
IRF5				
rs2004640G>T	NC_000007.13:g.128578301G>T<br>NM_001098629.1:c.-12+198G>T	T	Alternative splicing; ↑ *IRF5* expression	0.95
rs2070197T>C	NC_000007.13:g.128589000T>C<br>NM_002200.1:c.*128T>C	C	Intronic; regulatory; ↑ IRF5	0.02
rs10488631T>C	NC_000007.13:g.128594183T>C	C	3′ regulatory; ↑ *IRF5* expression	0.03
rs12539741C>T	NC_000007.13:g.128596805C>T<br>NM_012470.3:c.*31+473G>A	T	↑ IRF5	0.02
rs3807306G>T	NC_000007.13:g.128580680G>T<br>NM_032643.3:c.-11-1445G>T	T	TF binding modulation; ↑ IRF5	0.32
STAT4				

rs7574865T>G	NC_000002.11:g.191964633T>G NM_001243835.2:c.274-23582A>C	T	Intronic; ↑ *STAT4* expression	0.14
rs10181656G>C	NC_000002.11:g.191969879G>C NM_003151.3:c.274-28828C>A	G	↑ *STAT4* expression	0.14
rs13017460A>G	NC_000002.11:g.191932556A>G NM_003151.3:c.545-1315T>C	A	↑ *STAT4* expression	0.84
rs1517352A>C	NC_000002.11:g.191931464A>C NM_003151.3:c.545-223T>G	C	Intronic; ↑ *STAT4* expression	0.13
rs3821236G>A	NC_000002.11:g.191902758G>A NM_003151.3:c.1434+1167C>T	A	↑ *STAT4* expression	0.27
rs11889341C>T	NC_000002.11:g.191943742C>T NM_003151.3:c.274-2691G>A	T	↓ repressor activity leading to ↑ *STAT1* expression	0.13
rs7568275G>C	NC_000002.11:g.191966452G>C NM_003151.3:c.274-25401C>G	G	↑ *STAT4* expression	0.19
rs7582694C>G	NC_000002.11:g.191970120C>G NM_003151.3:c.274-29069G>C	C	↑ *STAT4* expression	0.17
TNFAIP3				
rs5029939C>G	NC_000006.11:g.138195723C>G NM_001270508.1:c.296-259C>G	G	Regulatory; ↓ *TNFAIP3* expression	0.36
rs2230926T>G (F127C)	NC_000006.11:g.138196066T>G NM_001270508.2:c.380T>G NP_006281.1:p.Phe127Cys	G (127C)	Missense (loss-of-function); ↓ A20 activity	0.35
rs6922466A>G	NC_000006.11:g.138444930A>G	G (protective)	↓ *TNFAIP3* expression	0.23
rs13192841G>A	NC_000006.11:g.137967214G>A	A (protective)		0.17
rs5029953G>A (Tags A125V in African pop.s)	NC_000006.11:g.138200760G>A NM_001270508.1:c.1906+272G>A	G	↓ TNFAIP3 deubiquitinating activity	0.87
rs5029941C>T (A125V)	NC_000006.11:g.138196060C>T NM_001270508.2:c.374C>T NP_006281.1:p.Ala125Val	T (125V; protective)	↓ TNFAIP3 deubiquitinating activity	0.02
BANK1				
rs10516487G>A (R61H)	NC_000004.11:g.102751076G>A NM_017935.5:c.182G>A NP_060405.5:p.Arg61His	G (R61)	↑ BANK1 *activity;* hyperactive autoantibody production	0.76
rs17266594 T>C	NC_000004.11:g.102750922T>C NM_017935.4:c.71-43T>C	T	eQTL; ↑ *BANK1* expression	0.75
rs3733197G>A (A383T)	NC_000004.11:g.102839287G>A NM_017935.5:c.1147G>A NP_060405.5:p.Ala383Thr	G (A383)	Missense; B-cell hyperactivity	0.81

rs4699261G>A	NC_000004.11:g.102725474G>A NM_017935.4:c.70+13367G>A	A	Intronic; ↑ *BANK1* expression	0.18
BLK				
rs13277113G>A	NC_000008.11:g.11491677G>A	A	Promoter; ↓ *BLK* transcription	0.14
rs2736340C>T	NC_000008.10:g.11343973C>T	T	Promoter; ↓ *BLK* transcription	0.14
rs2618476T>C	NG_023543.2:g.6021T>C NM_001715.2:c.-2+441T>C	C	-	0.14
ITGAM				
rs1143679G>A; (R77H)	NC_000016.9:g.31276811G>A NM_001145808.2:c.230G>A NP_001139280.1:p.Arg77His	A (77H)	Missense; ↓ *ITGAM* RNA; ↓ receptor function	0.10
rs9888739C>T	NC_000016.9:g.31313253C>T NM_001145808.1:c.1710+3978C>T	T	↓ *ITGAM* expression (LD with R77H)	0.55
rs1143683C>T (A858V)	NC_000016.9:g.31336888C>T NM_001145808.2:c.2576C>T NP_001139280.1:p.Ala859Val	C (858V)	Non-synonymous; CD11b receptor dysfunction	0.18
FcγR2A				
rs1801274A>G (H131R)	NC_000001.10:g.161479745A>G NM_001136219.3:c.500A>G NP_001129691.1:p.His167Arg	G (131R)	Missense; Altered FcγR2A receptor with ↓ binding affinity	0.54
FcγR3A				
rs396991A>C (F176V)	NC_000001.10:g.161514542A>C NM_000569.8:c.526T>G NP_000560.7:p.Phe176Val	A (F176)	Non-synonymous; ↓ IgG binding affinity	0.72
